# A STUDY ON JOB STRESS AS A MEDIATOR BETWEEN DEMOGRAPHIC VARIABLES AND SELF-REPORTED HEALTH AMONG OLDER ADULTS

**DOI:** 10.1093/geroni/igad104.3058

**Published:** 2023-12-21

**Authors:** Madhyami Deshmukh, Peter Martin

**Affiliations:** Iowa State University, Ames, Iowa, United States; Iowa State University, Ames, Iowa, United States

## Abstract

Job stress among older adults has not been sufficiently researched, and findings balance between work and personal life has become imperative. The aim of this research is to examine a path model that includes demographic variables (age, gender, and education), job stress, and self-reported health. Two recent waves (waves 13 and 14) of the Health and Retirement Study (HRS) were included in this analysis. A total of 8535 participants (3971 males and 4564 females) were part of this study. Bivariate correlations and multiple regression analyses were computed. The process macro in SPSS was used to assess mediation. Results indicated that job stress was associated with age, gender, and education such that women and more highly educated participants had higher job stress levels, whereas older adults had lower job stress levels. The multiple regression analysis showed that only age was significantly associated with stress, whereas gender and education were not. Furthermore, education was positively associated with better health, but health did not correlate with age or gender. As expected, job stress was associated with self-reported health at a later time point, such that more job stress was related to lower self-reported health levels. Finally, job stress mediated between demographic variables and health at a later time point. As the data were collected before the pandemic, it would be important to see if these results would have changed over the course of the pandemic. Future interventions should develop appropriate work-life balance to promote better health in later life.

